# Impact of image quality on OCT angiography based quantitative measurements

**DOI:** 10.1186/s40942-017-0068-9

**Published:** 2017-05-15

**Authors:** Mayss Al-Sheikh, Khalil Ghasemi Falavarjani, Handan Akil, SriniVas R. Sadda

**Affiliations:** 1 Doheny Image Reading Center, Doheny Eye Institute, Los Angeles, CA USA; 2 Department of Ophthalmology, David Geffen School of Medicine, University of California – Los Angeles, Los Angeles, CA USA; 3 Department of Ophthalmology, University Hospital Zurich, University of Zurich, Zurich, Switzerland; 4 Eye Research Center, Rassoul Akram Hospital, Iran University of Medical Sciences, Tehran, Iran

**Keywords:** OCT angiography, Image quality, Quantitative analysis, Image artefacts

## Abstract

**Background:**

To study the impact of image quality on quantitative measurements and the frequency of segmentation error with optical coherence tomography angiography (OCTA).

**Methods:**

Seventeen eyes of 10 healthy individuals were included in this study. OCTA was performed using a swept-source device (Triton, Topcon). Each subject underwent three scanning sessions 1–2 min apart; the first two scans were obtained under standard conditions and for the third session, the image quality index was reduced using application of a topical ointment. En face OCTA images of the retinal vasculature were generated using the default segmentation for the superficial and deep retinal layer (SRL, DRL). Intraclass correlation coefficient (ICC) was used as a measure for repeatability. The frequency of segmentation error, motion artifact, banding artifact and projection artifact was also compared among the three sessions.

**Results:**

The frequency of segmentation error, and motion artifact was statistically similar between high and low image quality sessions (P = 0.707, and P = 1 respectively). However, the frequency of projection and banding artifact was higher with a lower image quality. The vessel density in the SRL was highly repeatable in the high image quality sessions (ICC = 0.8), however, the repeatability was low, comparing the high and low image quality measurements (ICC = 0.3). In the DRL, the repeatability of the vessel density measurements was fair in the high quality sessions (ICC = 0.6 and ICC = 0.5, with and without automatic artifact removal, respectively) and poor comparing high and low image quality sessions (ICC = 0.3 and ICC = 0.06, with and without automatic artifact removal, respectively).

**Conclusions:**

The frequency of artifacts is higher and the repeatability of the measurements is lower with lower image quality. The impact of image quality index should be always considered in OCTA based quantitative measurements.

## Background

Optical coherence tomography angiography (OCTA) is a recently developed clinical tool that has allowed a non-invasive technique to visualize the retinal and choroidal microcirculation in a depth-resolved fashion allowing the superficial and deep retinal layer to be studied separately without the need for dye injection [1, 2]. This technology relies on motion contrast to separate moving from stationary structures to identify blood flow. OCTA has shown its ability to demonstrate pathological changes in various retinal and choroidal diseases including diabetic retinopathy, retinal vascular occlusions, macular telangiectasia, and choroidal neovascularization. Various quantitative metrics such as vessel density (VD) as caliber per area, length per area, and fractal dimension have been reported for the analysis of OCTA images. Many groups including ours have shown that the superficial and deep capillary circulation can be quantified reliably [3–5]. Despite these many advantages, the interpretation of OCTA images may be affected by various types of artifacts as well as by image quality. These artifacts may affect the accuracy of the measurements [6–8]. Previous studies have reported various cutoff values for signal strength without further investigation of its influence on quantitative measurements [9–11]. To the best of our knowledge, no study has reported the effect of image quality on quantitative analysis of OCTA images. The aim of this study was to investigate and to quantify the impact of image quality on OCTA quantitative measurements.

## Methods

This prospective comparative study was approved by the Institutional Review Board of the University of California Los Angeles and conducted in accordance with the ethical standards stated in the Declaration of Helsinki. The study was carried out in accordance with Health Insurance Portability and Accountability Act regulations. All subjects gave their written informed consent after the purpose of the study had been adequately explained. Healthy individuals older than 18 years with no previous history of ophthalmologic or systemic diseases were recruited from the Doheny Eye Center, University of California - Los Angeles. Any evidence of pathology on clinical examination or structural OCT was grounds for exclusion. Patients with any visual complaints, refractive error greater than 2.5 diopters, history of surgical intervention (including refractive surgery) were also excluded.

### Swept-source OCT-angiography

All OCTA scans were performed by a single experienced examiner using a swept-source OCT device (DRI OCT Triton, TOPCON Inc, Tokyo, Japan). The device operates with a central wavelength of 1050 nm, an acquisition speed of 100,000 A-scans per second, and an axial and transverse resolution of 7 and 20 μm in tissue. The scans were taken from a 3 × 3 mm cube, with each cube consisting of 320 clusters of four repeated B-scans centered on the fovea. To investigate repeatability of the measurements, two sets of scans were obtained for each subject, 1–2 min apart. To investigate the impact of image quality on quantitative measurements we applied a lubricating gel (GenTeal, Alcon Laboratories, Inc., a Novartis Company, Fort Worth, Texas, USA) after the second session and immediately repeated the same scan protocol. A good image quality is more than 40 according to the OCT manufacturer.

**Fig. 1:**
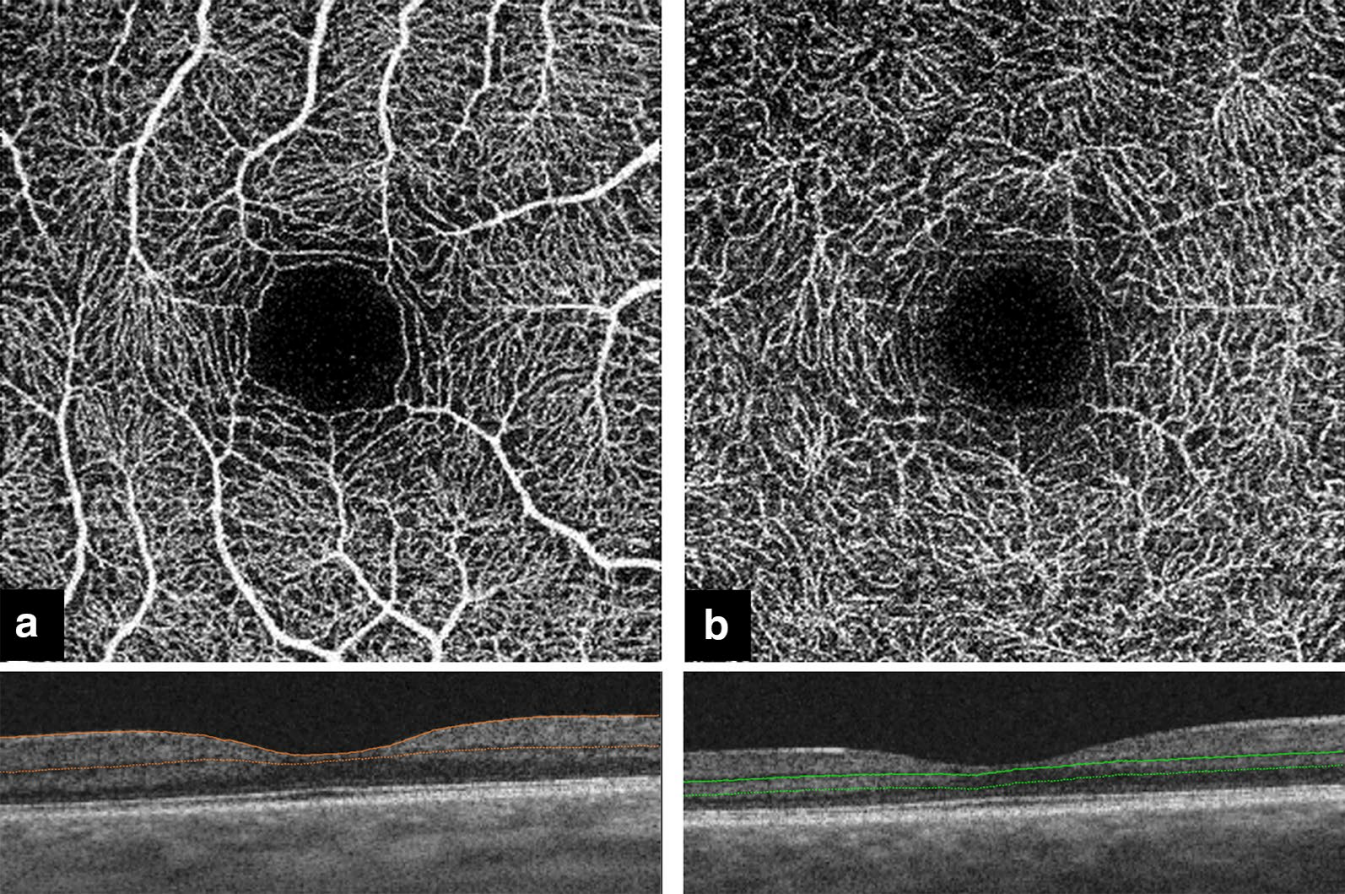
Automated segmentation of the retinal layers. **a** The superficial retinal layer (SRL) with the corresponding B scan. The SRL extends from 2.6 μm beneath the internal limiting membrane to 15.6 μm beneath the interface of the inner plexiform layer and inner nuclear layer (IPL/INL). **b** The deep retinal layer (DRL) with the corresponding B scan. The DRL slab was generated from 15.6 μm beneath the IPL/INL to 70.2 μm beneath IPL/INL

En face images were generated from the superficial retinal layer (SRL) and deep retinal layer (DRL) based on automated layer segmentation performed by the OCT instrument software (IMAGEnet 6 V.1.14.8538) (Fig. 1). The automated segmentation defines the en face slab for the SRL to extend from 2.6 μm beneath the internal limiting membrane to 15.6 μm beneath the interface of the inner plexiform layer and inner nuclear layer (IPL/INL). The DRL slab was generated from 15.6 μm beneath the IPL/INL to 70.2 μm beneath IPL/INL. The centration of the fovea was checked for all images. All images were reviewed for the segmentation errors for the SRL and DRL according to the previously described method and the frequency of segmentation error was compared among the three sessions [6]. Additional image artifacts including motion, banding and projection artifacts were evaluated as well.

### Quantitative measurements and statistical analysis

For this study, en face slabs of the SRL and DRL were used for quantitative analyses. The quantitative analysis was performed using the publically available ImageJ software (public domain software, National Institutes of Health, Bethesda, Maryland, USA) [12]. The DRL en face image was analyzed with and without using OCT device’s proprietary artifact removal tool. The VD was expressed as a ratio by taking the total vessel area divided by the total area of the analyzed region in the entire 3 × 3 mm scan as previously described [13]. After extracting the original images from the viewing software, the images were then imported into ImageJ. For the VD measurement, we used a binarized image with intensity thresholding with Otsu’s thresholding method as implemented in ImageJ. Otsu’s method assumes that the image contains two classes of pixels following a bi-modal distribution. It calculates optimum threshold by minimizing intraclass variance and maximizing interclass variance [12]. The total number of pixels occupied by vessels was then divided by the total number of pixels from the entire image and the value was expressed as a ratio.

All statistical analyses were performed using SPSS software version 21 (SPSS, Inc., Chicago, IL). Paired samples were tested for normal distribution using the Shapiro Wilks test. Chi square test and Fisher’s exact test were used to compare the categorical parameters. Paired t test was used to compare the continuous variables. The first session with the higher image quality index was selected for comparison between high and low image quality measurements. The intraclass correlation coefficient (ICC) was used to analyze repeatability. The analysis for all significant differences was repeated to account for the correlation between two eyes of participant that had both eyes included. For this purpose, one eye of each participant was randomly selected. A P value of <0.05 was considered significant.

## Results

Seventeen eyes of 10 patients with a mean age of 37 ± 5.8 years (range 26–45) were studied. The image quality index was 72, 73.18, and 52.29 for the first, 2nd and 3rd session, respectively (P = 0.163 for 1st and 2nd sessions and P < 0.001 for 1st and 3rd session).

**Fig. 2:**
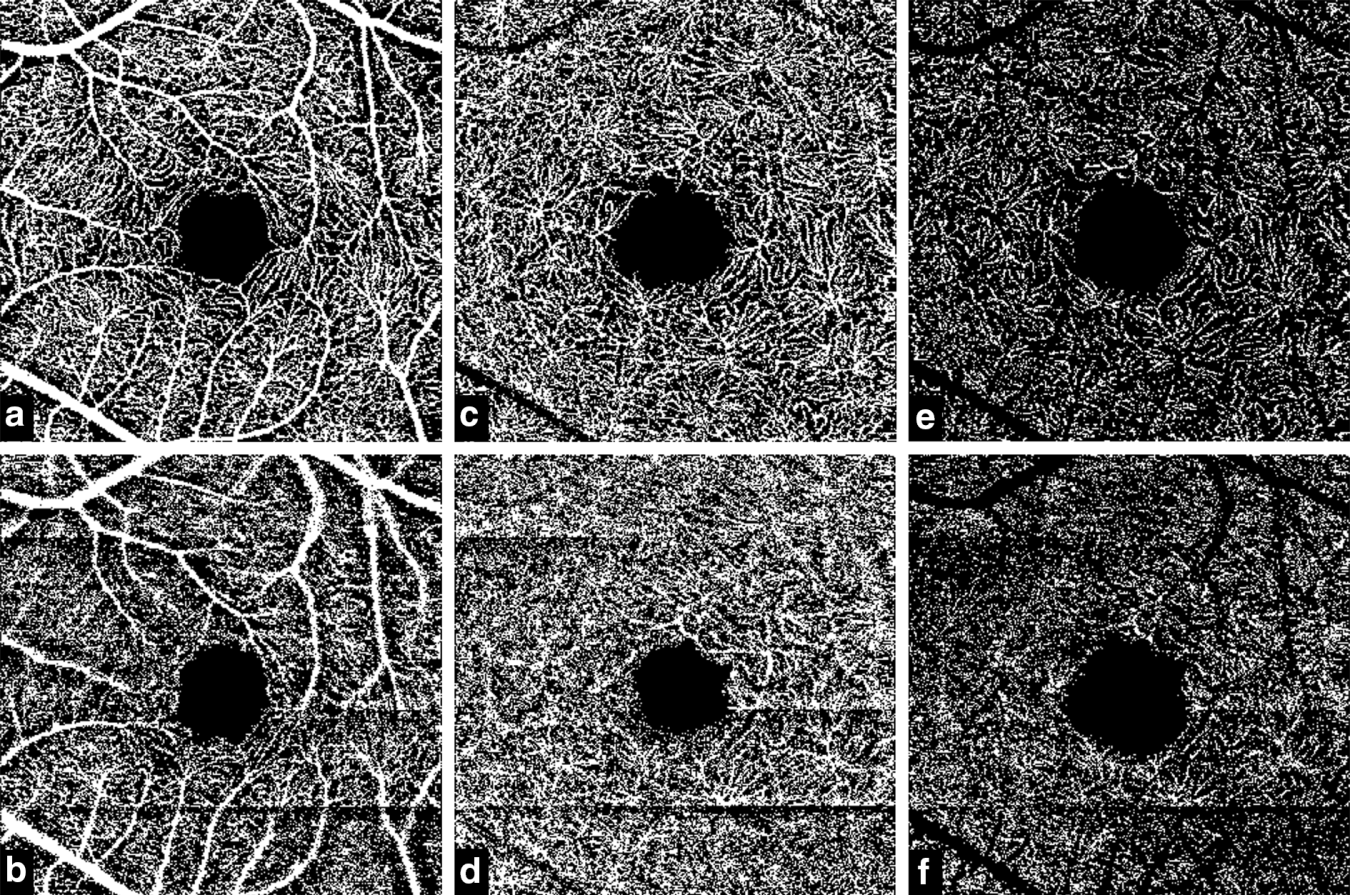
OCT Angiography en face images of the superficial and deep retinal layer. **a** The binarized en face image of the superficial retinal layer (SRL) with high Image quality. **b** The SRL image with reduced image quality. **c**, **d** The corresponding deep retinal layer (DRL) without using the artefact removal tool, **e**, **f** the corresponding DRL with the artefact removal tool

On qualitative inspection, the largest-sized vessels appeared thicker and the medium and small sized vessel were not completely visible in the low quality images compared to the high quality images (Fig. 2). In the DRL, the vessels appeared thicker in the low quality image compared to the high quality images.

The values of the VD measurements for the first, second and third session with reduced image quality index (IQI) are presented in Table 1.

**Table 1:** Vessel density (ratio) of the superficial and deep retinal layer in 3 different sessions

	1st session	2nd session	3rd session	IQI		P value	ICC
SRL	0.408 ± 0.012	0.410 ± 0.011	0.397 ± 0.020	72	1st vs 2nd session	0.4	0.807
					1st vs 3rd session	0.04	0.338
DRL^a^	0.394 ± 0.022	0.383 ± 0.014	0.447 ± 0.028	73.18	1st vs 2nd session	0.064	0.525
					1st vs 3rd session	<0.001	0.063
DRL^b^	0.132 ± 0.007	0.128 ± 0.008	0.148 ± 0.014	52.29	1st vs 2nd session	0.071	0.660
					1st vs 3rd session	<0.001	0.368

*SRL* superficial retinal layer, *IQI* image quality index, *ICC* intraclass correlation coefficient

^a^ *DRL* deep retinal layer without artefact removal

^b^ *DRL* deep retinal layer with artefact removal

Segmentation errors, which were uncommon, were similar between the different sessions with the presence of segmentation error in one eye in the first and second session (P = 1), and 2 eyes in the session with reduced IQI (P = 0.707). The segmentation error was limited to 1–3 B-scans and was deemed to be unlikely to affect the global VD measurements. Motion artifacts were present in one eye in the first and third session (P = 1); banding artifacts were present in 3 eyes in the first and second session and 12 eyes in the third session (P = 0.218). There was no projection artifact in the first session, one projection artifact in the second session (P = 0.797) and in 8 eyes in the third session (P = 0.274).

Quantitatively, the VD in the SRL was reduced with lower image quality, whereas it was higher in DRL with lower image quality. By ICC analysis, there was a high level of agreement in SRL VD between the first two sessions but a poor agreement between the first and the third session. For the DRL, the agreement was moderate between the two high quality sessions, but poor between the first (high quality) and third (low quality) session.

To confirm that there was no bias introduced by the few cases where both eyes from the same subject were included, the analysis was repeated after randomly choosing only one eye for each subject, and the same level of agreement and significance was observed for all measurements.

## Discussion

In this study, we evaluated the impact of IQI on OCTA based quantitative measurements as well as the impact on image artifacts. We found that the frequency of projection and banding artifacts was higher and the repeatability of the vessel density measurements was lower after reducing the image quality. Previous studies have shown that several factors including imaging technique and processing software, patient characteristics, and ocular pathologies may lead to significant error in OCT measurements [14–19]. However, our study is the first to show that image quality affects the frequency of errors in OCTA measurements. We applied topical eye gel to simulate media opacity and reduce the image quality. Degrading image quality appears to affect the accuracy of the automated software algorithms, and these errors result in variance of the VD measurements. Although the manufacturer recommended the image index of higher than 40 is good, our results show that rate of artifacts is higher with the lower image quality, even in the manufacturer recommended range.

Gao et al. [20] applied a reflectance-adjusted threshold for flow detection to improve the reliability of vessel density measurements compared to the conventional method of using a fixed threshold. The neutral density filters were used to change the image reflectance in five healthy participants. They reported that reflectance compensation reduced population variation in 25 healthy eyes from 8.5 to 4.8% (coefficient of variation) in the 6x6 images from macula, highlighting the importance of reflectivity or signal variation on the repeatability and reliability of quantitative vessel metrics from OCTA images.

Several studies have reported VD measurements in healthy eyes and eyes with different ocular diseases [3–5, 9, 13, 21–24]. Other studies have shown the correlations of the VD measurements with other image measurement modalities [25–27]. Although some studies reported that images with good quality images were selected for the analysis, the impact of image quality index has not been reported. Considering the effect of low image quality on the qualitative and quantitative analysis of the vessel density, we would recommend that the image quality factor or its equivalents (e.g. signal to noise ratio, signal strength index, etc.) should be incorporated when the patient information is presented.

Our study is one the first investigations to describe the impact of image quality on quantitative measurements by OCTA and has several strengths which should be noted. First, the data was collected in a prospective fashion with a standardized protocol. Moreover, to evaluate the impact of image quality, we performed repeat scans to assess measurement repeatability and demonstrated a high level of agreement between sessions. Finally, we used a swept-source OCTA device with a 1050 nm light source wavelength, with less sensitivity roll-off and better penetration through media opacities. Further studies may be necessary to better define the magnitude of impact of image quality on conventional spectral domain OCT devices—we might expect an even bigger impact in that situation.

Our study is also not without limitations. The sample size is small and we did not include eyes with various ocular pathologies. We also measured the VD using binarization of the images, which is a technique implemented by many OCTA manufacturers. Skeletonization of the binarized images is a different method for the measurement of vessel density. Skeletonization of the vessels causes the width of all vessels to be reduced to a single pixel. Therefore, vessels, whether originally narrow or wide, are reduced to lines of similar width. Image quality may affect the vessel width (e.g. “blurry” vessels may appear wider), consequently, skeletonization of the vessels may reduce this variability.

In conclusion, our study shows that the quality of the image affects the frequency of errors and vessel densities on OCTA. Further studies with larger sample size and inclusion of eyes with ocular pathologies are needed to confirm our findings.

## Abbreviations

OCTA: optical coherence tomography angiography
IQI: image quality index
SRL: superficial retinal layer
DRL: deep retinal layer
ICC: intraclass correlation coefficient
VD: vessel density
